# Supplementary material for: Sub-8 nm networked cage nanofilm with tunable nanofluidic channels for adaptive sieving
Source: Nat Commun. 2024 Mar 20;15:2478. doi: 10.1038/s41467-024-46809-4 (PMC10954766; doi:10.1038/s41467-024-46809-4)
Supplement: Supplementary file 1 — Supplementary Information [file 41467_2024_46809_MOESM1_ESM.pdf]

Supplementary Information

Sub-8 nm Networked Cage Nanofilm with Tunable Nanofluidic Channels for Adaptive Sieving

Si-Hua Liu[1,4], Jun-Hao Zhou[1,4], Chunrui Wu[2], Peng Zhang[3], Xingzhong Cao[3], Jian-Ke Sun[1]*

[1] MOE Key Laboratory of Cluster Science, Beijing Key Laboratory of Photoelectronic/Electrophotonic Conversion Materials, School of Chemistry and Chemical Engineering, Beijing Institute of Technology, Beijing 102488, P. R. China;

[2] State Key Laboratory of Separation Membranes and Membrane Processes, School of Chemical Engineering and Technology, Tiangong University, Tianjin 300387, P. R. China;

[3] Key Laboratory of Nuclear Analysis Techniques, Institute of High Energy Physics, Chinese Academy of Sciences, Beijing 100049, P. R. China

[4] These authors contributed equally: Si-Hua Liu, Jun-Hao Zhou.

[*] Correspondence author. Email: jiankesun@bit.edu.cn (J.-K. Sun).

This PDF file includes:

Supplementary Methods

Supplementary Figures 1 to 36

Supplementary Tables 1 to 4

Supplementary References

Supplementary Methods

1 Membrane performance evaluation

1.1 Water permeance and solute rejection

The separation performance of the cage composite membranes was evaluated in a dead-end stirred cell. The effective area of the membrane test cell is 3.14 cm^2. Operating pressure was fixed at 1.0 bar. To ensure consistent results, all performance data were collected after a pre-compaction process of 30 minutes. Concentrations of the solutes in the feed solutions were 100 mg L^{-1}. Pure water permeance (J_w, L m^{-2} h^{-1}) and rejection (R, %) were calculated by the following equations:

$$J_w = \frac{V}{S \cdot t} \quad (1)$$

where V, S, and t are the volume of permeated water (L), the valid membrane filtration area (m^2), and the permeation time (h), respectively.

$$R = \left(1 - \frac{C_p}{C_f}\right) \times 100\,\% \quad (2)$$

where C_f and C_p (mg L^{-1}) are the solute concentration of the feed and permeate solution, respectively, which were measured with an Ultraviolet–visible spectroscopy. Measurements were performed on at least three samples for each membrane, and the average values were reported.

1.2 Water transport calculation

We calculated the intrinsic water permeability (P_w, cm^2 s^{-1}) of the networked cage nanofilms using the solution-diffusion model to compare their water transport capability with traditional polymeric membranes and other emerging nanofluidic membranes. Figure 2f in the main text shows the comparison of P_w values between the cage composite membranes and traditional polymeric membranes. The raw data used for this analysis were collected from previously published papers.

The water permeance through the composite membrane is given as follows:

$$J_w = A(\Delta p - \Delta \pi) \quad (3)$$

Where A (L m^{-2} h^{-1} bar^{-1}) is the water permeance constant, Δp (bar) and $\Delta \pi$ (bar) are the differences in hydraulic pressure and osmotic pressure across the membrane, respectively. $\Delta \pi$ was calculated by the following equations:

$$\Delta \pi = (C_f - C_p)RT \quad (4)$$

The constant A and P_w are related by the following equation:

$$A = \frac{P_w}{L}\frac{M_w}{RT} \quad (5)$$

Where M_w is molecular weight of water (g mol^{-1}), L is membrane thickness (cm), R is gas constant (83.1 cm^3 bar mol^{-1} K^{-1}) and T is absolute temperature (K).

1.3 Stability performance measurement

The crosslinked amine cage nanofilm is expected to exhibit better chemical stability in comparison to the packed cage nanofilm. To confirm this, membrane samples were immersed in acid solutions (HCl, pH=3) and polar (methanol) /nonpolar (hexane) organic solvents for 60 min. Water permeance and Congo red rejection were recorded before and after these treatments, and the normalized water permeance and rejection values were reported. For comparison, composite membrane prepared by spin-coating of the Cage 1 were underwent the same process of chemical stability evaluation.

A 120-hours continuous filtration test was conducted using a commercial cross-flow filtration apparatus. The operating pressure and cross-flow rate were fixed at 1.0 bar and 50.0 L h^{-1}, respectively. The temperature of the feed solutions (Congo red, 100 mg ml^{-1}) was controlled at 25 ± 0.5 °C by a heat exchanger.

In addition, the water flux and Congo red rejection of the prepared membranes were measured under applied pressure ranging from 1.0 to 5.0 bar to estimate the robustness of the networked cage nanofilms.

1.4 Graded separation

Three-component graded sieving dye separations were carried out using 4-nitrophenol (4-NP, 2.5 Å), methyl orange (MO, 4.8 Å), and Congo red (CR, 5.2 Å) dissolved in water. The experimental procedure involved the following steps: 1) 50 mL of an aqueous feed containing 20 mg L^{-1} of the 4-NP, MO, and CR dyes was added to the dead-end stirred cell. The first-round filtration was operated after UV-irradiation. Only 4-nitrophenol was detected in the permeate, and Congo red and methyl orange were nearly completely rejected. After Vis irradiation, the methyl orange became permeable while the Congo continued to be rejected. After flushing the residual methyl orange from the feed with excess deionized water, the pure phase of Congo red could be collected. The set-up used for the light controlled graded molecular separation is presented in Supplementary Fig. 1.

2 Characterization methods

2.1 Nuclear magnetic resonance

^{1}H nuclear magnetic resonance (^{1}H-NMR) was measured by a Bruker DPX-400 spectrometer operating at 400 MHz and room temperature. Prior to the NMR measurement, all solutions were filtrated with a syringe filter (pore size: 0.22 μm) to

remove the insoluble impurities.

2.2 Electrospray mass spectroscopy

Electrospray ionization mass spectrometry (ESI-MS) was employed to determine the molecular weight of the dissolved organic cage in the aqueous solution. This measurement was performed on an Agilent Q-TOF 6520 spectrometer. Ultrapure water was used to prepare the aqueous solution, which was further filtrated with a syringe filter (pore size: 0.22 μm) to remove the insoluble impurities.

2.3 Fourier transform infrared spectroscopy

The crosslinked chemical structure of the cage nanofilms was preliminarily verified by Fourier transform infrared (FT-IR) spectra, which were collected on a Nicolet iS10 FT-IR spectrometer. Composite membrane samples were cut into size and recorded in transmission mode using 32 scans with a resolution of 4 cm^{-1}.

2.4 X-ray photoelectron spectroscopy

Details in atomic composition of the networked cage nanofilms were analyzed by a PHI 5000 Versa probe III X-ray photoelectron spectrometer using Al Kα radiation. To compensate for surface charging effects, all XPS data were referenced to the C1*s* neutral carbon peak at 284.6 eV. Two layers of the cage nanofilm were transferred to the silicon wafer disc to conduct the XPS analysis.

As nitrogen element only comes from the cage molecule, we can calculate the percentage of crosslinked -NH- (PC_{NH}) in the amine cage molecule by following equation:

$$PC_{NH} = S_{O=C-N}/(S_{NH} + S_{N^+} + S_{O=C-N}) \quad (6)$$

where $S_{O=C-N}$, S_{NH} and S_{N^+} were the peak area of O=C-N, -NH-, and -N^+- components in N1*s* narrow scan, respectively. The PC_{NH} was calculated to be ~70 %. Because the oxygen element only comes from the trimesoyl chloride (TMC) molecule while the nitrogen element only comes from the cage molecule, we can further analyze the crosslinked structure in the cage nanofilm according to the following method:

$$\frac{O}{N} = 3X/12Y \quad (7)$$

Where X and Y represent the number of TMC ring and cage skeleton in the cage nanofilm, respectively. The ratio of X/Y was calculated to be ~7.9. On average, each cage molecule binds to other four cages through acyl chloride bridges.

2.5 Scanning electron microscopy and energy dispersive spectrometer

Surface morphologies of the porous supports and composite membranes were observed by a field emission scanning electron microscopy (FESEM, JSM-7500F, JEOL, Japan)

with an accelerating voltage of 5 kV. Before observation, all the samples were sputtered with platinum particles with a radius of ~5 nm. Energy dispersive spectrometer (EDS) analysis was conducted using the same machine with an accelerating voltage of 15 kV.

2.6 Atomic force microscope

Atomic force microscope (AFM, Icon, Bruker, United States) imaging was used to characterize the surface morphology and thickness of the cage nanofilms and other control samples. A sampling resolution of at least 256 points per line and a speed of 0.1-1.5 Hz were used. Software program NanoScope Analysis was used to process the AFM images. To perform the test, free-standing nanofilms were transferred to silicon wafers and dried. Edge of the nanofilm was purposely loaded on the silicon wafer to form the step.

2.7 Positron annihilation lifetime spectroscopy

The free volume characteristics of the networked cage powder were determined by high resolution positron annihilation lifetime spectroscopy (PALS, EG&G, USA) with a fast-slow coincidence system. Powder samples with dimensions of approximately 1 cm × 1 cm × 0.5 μm were prepared, and the positron source (^{22}Na, 13 μci) was placed between two identical samples. The detectors of PALS are a pair of BaF_2 probes with a resolution of 195 ps. Each spectrum was recorded at least 2×10^6 coincidences and resolved with the LT 9.0 program. The radius of the free volume cavities, r_3, was calculated according to the following equation:

$$\tau_3 = \frac{1}{2}\left[1 - \frac{r_3}{r_3+\Delta r} + \left(\frac{1}{2\pi}\right)\sin\left(\frac{2\pi r_3}{r_3+\Delta r}\right)\right]^{-1} \quad (8)$$

where τ_3 and Δ_r are the ortho-positronium (o-Ps) pickoff lifetime and the thickness of the electron layer (0.1656 nm), respectively.

2.8 Zeta potential

Surface charge of the cage composite membrane was determined by the streaming potential method using an electrokinetic analyzer (SurPASS 3, Anton Paar GmbH, Austria) with an electrolyte solution of 0.001 M potassium chloride (KCl), whereas 0.5 M hydrogen chloride (HCl) solution and 0.05 M potassium hydroxide (KOH) solution were used to adjusted pH values of the electrolyte solution (3 to 10). The streaming potential results were then calculated by its built-in software using Helmholtz-Smoluchowski equation to determine the membrane zeta potential.

2.9 Water contact angle

Contact angle for cage composite membrane with different counterions were measured with a contact angle analyzer (DSA30S, KRUSS, Germany) at ambient temperature. A droplet of 2 μL DI water was delivered onto a membrane surface using a micro-syringe,

and a static image of the droplet in equilibration with the membrane surface was taken. Contact angle was calculated with a circle fitting method by drop shape analysis software. For any given membrane type, contact angle measurements were performed for at least 15 different locations.

2.10 UV-vis spectroscopy

Solution UV−vis absorption measurements were recorded with a Mapada UV-3100 spectrometer. All concentrations of dyes aqueous were determined by the method of UV-vis spectroscopy. In addition, we also used this device to in situ monitor the interfacial diffusion process of the aqueous monomers (cage 1 and (*R,R*)-1,2-diaminocyclohexane) [1]. Typically, the oil/water phase interface was created by adding 1.4 mL of aqueous solution (8 mM) and pure n-hexane to the quartz cuvette, respectively. The kinetic mode in the UV probe software was employed to in-situ monitor changes in absorbance at the characteristic absorption wavelength, reflecting the interfacial diffusion of monomers. The sampling interval was set at 30 seconds, and the test duration spanned 120 minutes.

2.11 Solid UV spectroscopy

Solid UV-vis absorption measurements were carried out at room temperature by using a PE Lambda 900 spectrometer.

3 Simulations

All simulations were based on the following details. As for electrostatic potential (ESP), the structure of molecules using full geometry optimization and frequency analysis with Gaussian 09 program[2]. The Grimme's description correction D3[3] was essential in system with noncovalent interactions. The calculation was based on M062x[4] combined with 6-31G**. To ensure the stability of the geometry, harmonic vibrational frequencies were employed at the same theoretical level without negative frequencies. In addition, fragment optimization was performed in the liquid phase. The SMD[5] was chosen to mimic the solvent (water) effect in all calculations.

The atomistic structure of cage nanofilms with different counterions was constructed from cage and TMC with a mole ratio of 1:8. The atomistic structure of cage and TMC was optimized by density-functional theory (DFT) calculations using GFN-xtb. The DFT calculations were conducted with GFN1-xtb. The pore size distribution of the *iac*-cage nanofilm was analyzed by Zeo++[6].

Molecular dynamics (MD) simulations were carried out to investigate the water transport process using Gromacs 2020.4[7]. Generalized Amber Force Field (GAFF)[8] was used for all simulations. Parameters for cage and counter ions molecules were generated

with sobtop 1.0(dev3.1)[9]. However, its description of the -N=N- bond was poor, leading to spontaneous isomerization between Z and E forms. This problem has been previously spotted and corrected parameters were developed based on the DFT studies. Therefore, we have replaced default GAFF parameters for C-N-N-C and N-N-C-C torsional potentials with those proposed by Duchstein et al[10].

3.1 Electrostatic potential distribution and self-assembly at oil/water interface

Electrostatic potential (ESP) was employed to display the amphiphilicity of the partially protonated cage molecules. The details are described as follows: The electrostatic potential (ESP) on the van der Waals (VDW) surfaces (iso-density = 0.001 a.u.) of the cage fragment was based on its ground state electron density. The ESP was fitted from optimized geometry and wave function using Multiwfn[11] software and Visual Molecular Dynamics (VMD)[12].

Further, we simulated the self-assembly behavior of the amphiphilic cage molecules at a water-hexane interface. All molecules were placed in a 7*7*12 nm box. The cut-off for neighbor list of Verlet method that for short-range interactions is 1.2 nm in all calculations with periodic boundary conditions in all three directions. The modified Berendsen thermostat (V-rescale in GROMACS) was used for temperature control. Berendsen pressure bath was used for possible pressure control. The Particle mesh Ewald (PME) method was used for electrostatics.

The system was subjected to energy minimization (with steepest descent algorithm), NVT and NPT equilibration phases (each 6 ns long), and final production phase (12 s), from which the average density and other parameters were collected. Visualization of the simulation (figures and movie) were performed using VMD.

3.2 Water molecules transport simulation

The *iac*-cage nanofilm model was constructed following the 4-step process: (1) The crosslinked *iac*-cage was built with counterions of Cl^-. (2) The system energy minimization (with steepest descent algorithm); (3) NVT and NPT equilibration phases (each 6 ns long); (4) Final production phase (12 ns), from which the average density and other parameters were collected; (5) As for other counterions, Cl^- was replaced by $TFSI^-$, azo-trans and azo-cis. (6) repeat step (2-4), get all the *iac*-cage nanofilm models.

All molecules were placed in a 20*6.6*2.6 nm box. The 8 nm *iac*-cage nanofilm model was restricted by a harmony force in three directions during NP_xT MD simulation, and the force constant was 10000 kJ nm^{-1} mol^{-1}. The 8 nm vacuum layer and 4 nm vacuum layer were added to the left and right of *iac*-cage nanofilm model. Subsequently, water solution was filled in the 8 nm vacuum layer. The graphene was

added to both sides of all models to eliminate the periodic along the x-axis. 72 ns NVT non-equilibrium MD simulation at 297.15 K was performed on those models.

Supplementary Fig. 32 and 33 displayed the simulation setup for water transport through the networked cage nanofilm. The membrane was sandwiched by a water chamber on the left and a vacuum chamber on the right. Two impermeable graphene plates were placed on both sides and acted as pistons. The pressures on the left and right graphene plates were P_{left} = 600 bar and P_{right} = 0 bar, respectively, resulting in a pressure difference ΔP of 600 bar.

The operation pressure is calculated from the moving acceleration rate (a) of left plate[13], $P = \frac{N \cdot m \cdot a}{A}$, where N is the total number of atoms contained in the left plate, m is the mass, A is the cross section of moving graphene and P is the applied pressure (600 bar).

3.3 Depiction of window opening size variation with light-responsive counteranions

We offered a method to semi-quantitatively describe the variation in window opening size of the *iac*-cage-azo-nanofilm when exposed to alternating UV and Vis irradiation. Specifically, we utilize the cage window fragment and the azo molecule to create projected models and calculate the projected area with the help of photoshop. Additionally, simplified geometric illustrations are generated based on these models to provide a clear demonstration of the window opening size variation.

Supplementary Figures and Tables

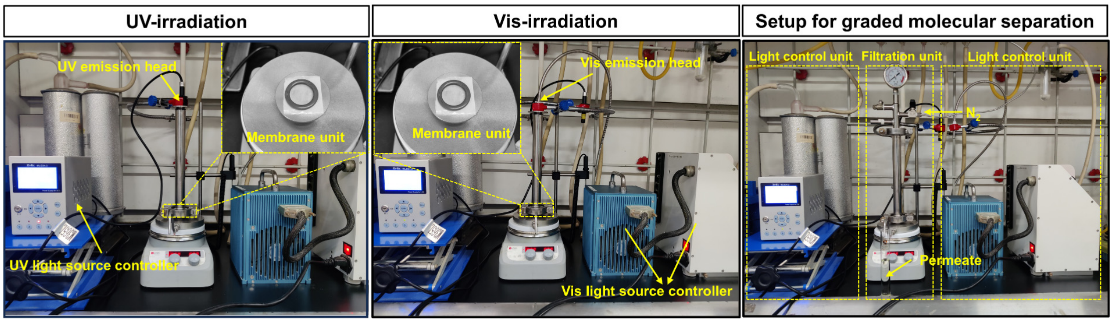

Supplementary Fig. 1 The set-up used for the light controlled graded molecular separation.

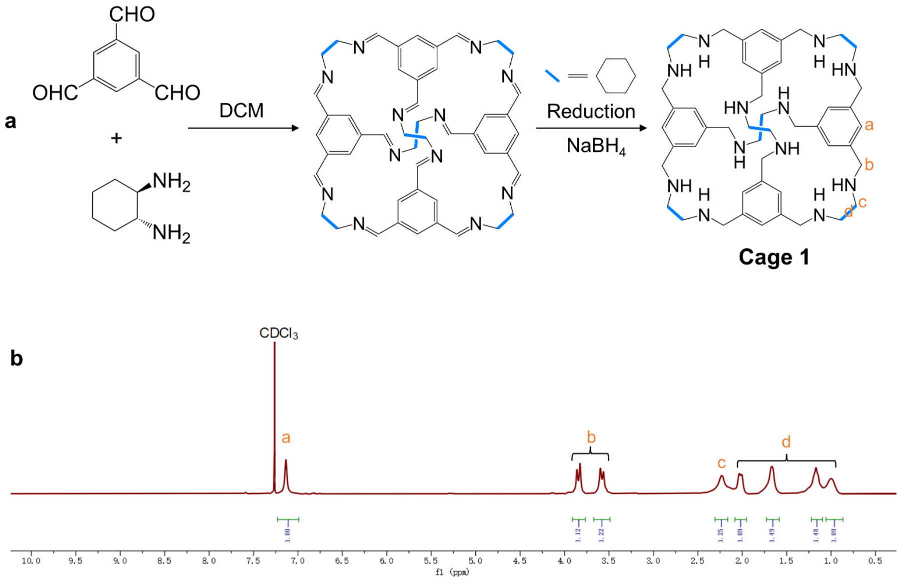

Supplementary Fig. 2 a) Synthetic procedure for Cage 1; b) The ¹H NMR spectrum of Cage 1 in $CDCl_3$.

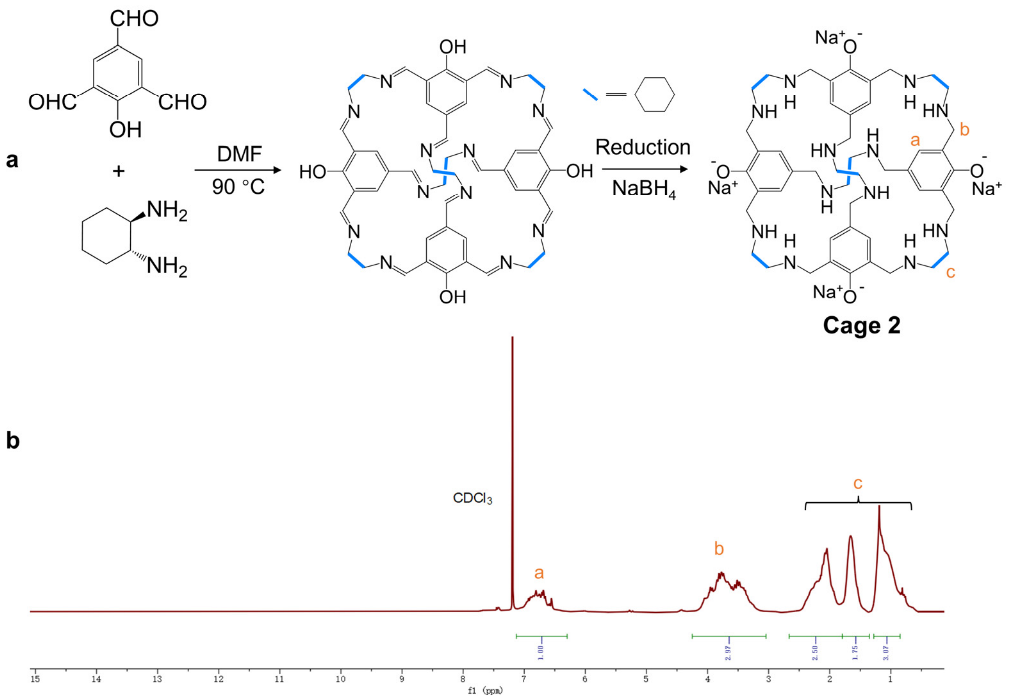

Supplementary Fig. 3 a) Synthetic procedure for Cage 2; b) The ^{1}H NMR spectrum of Cage 2 in $CDCl_3$.

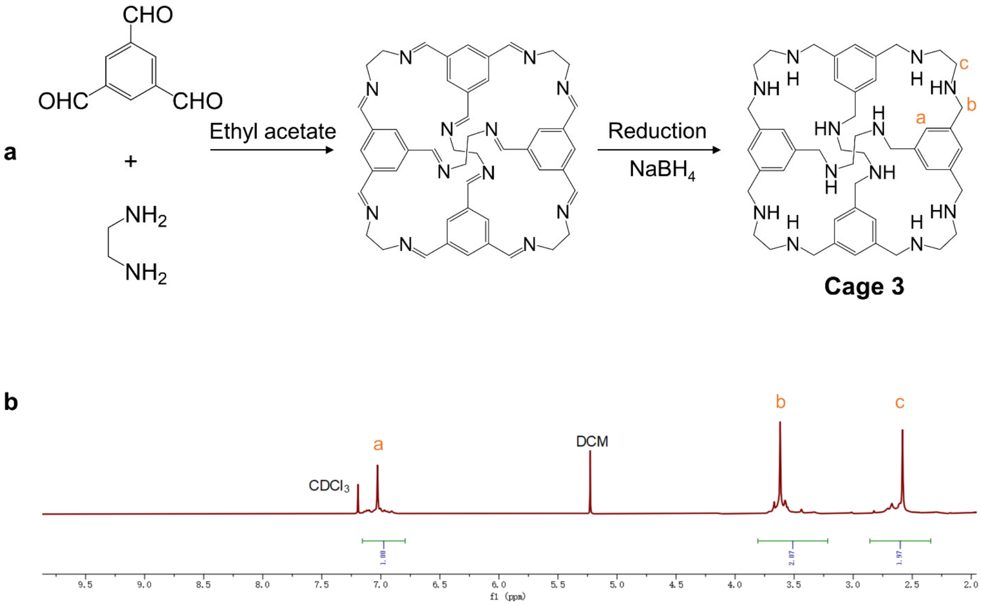

Supplementary Fig. 4 a) Synthetic procedure for Cage 3; b) The ^{1}H NMR spectrum of Cage 3 in $CDCl_3$.

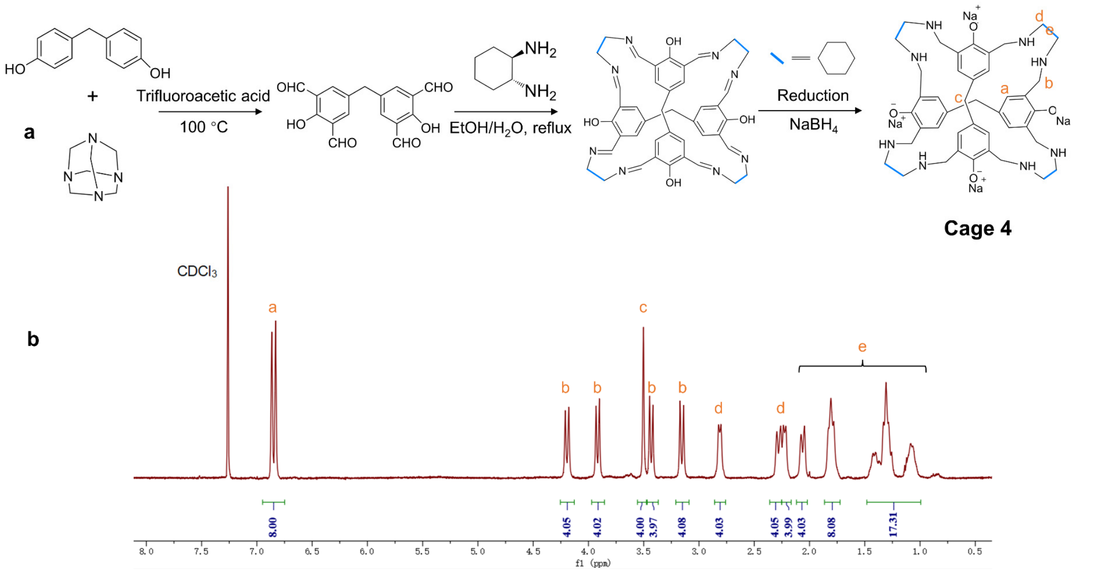

Supplementary Fig. 5 a) Synthetic procedure for Cage 4; b) The ^{1}H NMR spectrum of Cage 4 in $CDCl_3$.

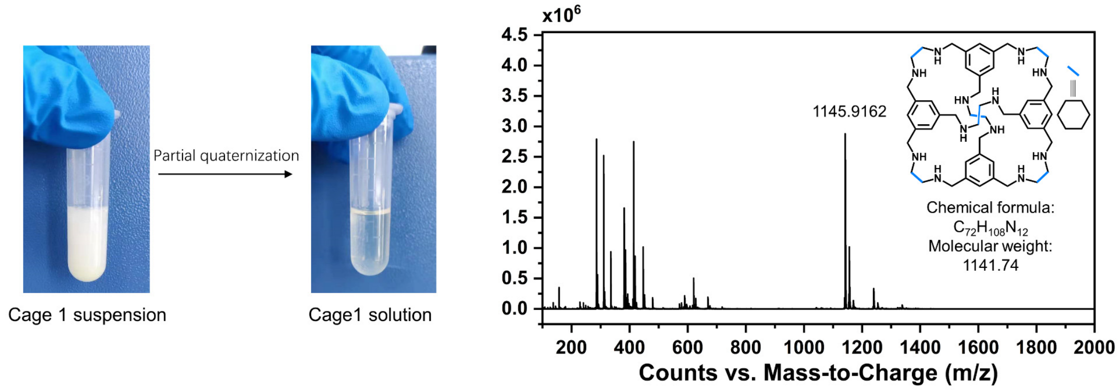

Supplementary Fig. 6 Electrospray ionization mass spectrometry of Cage 1 aqueous solution used for preparing the networked cage nanofilms. The selected main peak at m/z = 1145.9 corresponds to the cage with four protonated amine groups.

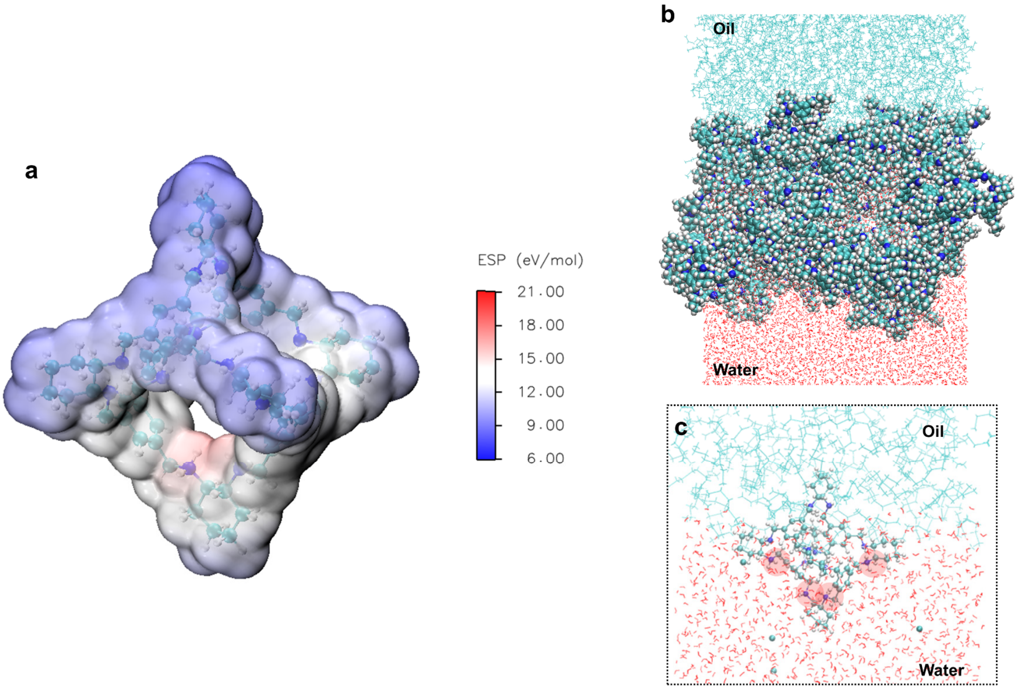

Supplementary Fig. 7 Self-assembly of the amphiphilic cage at oil-water interface. a) Electrostatic potential (ESP) mapping of the partial quaternized Cage 1 shows the amphiphilicity. b) The amphiphilic cage favors self-assembly at the sharp O/W interface and thus pre-organized into an ultrathin nanofilm. c) An amphiphilic cage 1 at the O/W interface with most amine groups inserted in the oil phase. The red circles highlight the protonated amine groups.

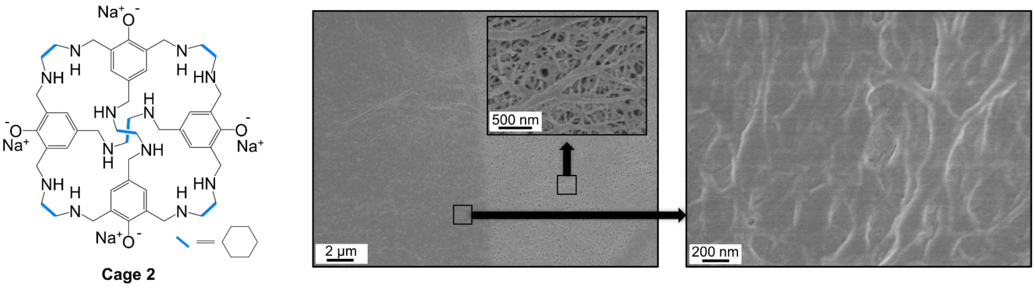

Supplementary Fig. 8 Top surface SEM image of nanofilm prepared from Cage 2. Synthesis conditions: **Cage 2** concentration: 4 mM; TMC concentration: 6 mM, Reaction time: 10 min.

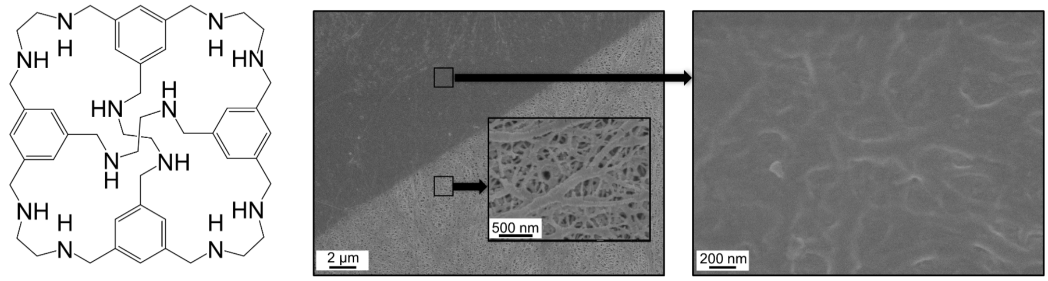

Cage 3

Supplementary Fig. 9 Top surface SEM image of nanofilm prepared from Cage 3. Synthesis conditions: **Cage 3** concentration: 4 mM; TMC concentration: 6 mM, Reaction time: 10 min.

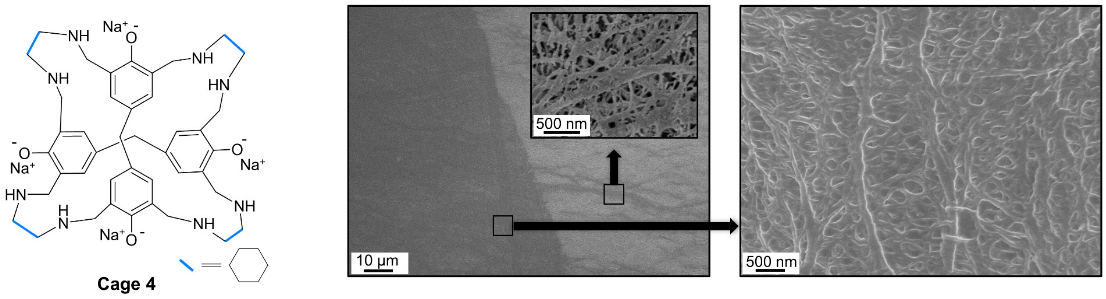

Cage 4

Supplementary Fig. 10 Top surface SEM image of nanofilm prepared from Cage 4. Synthesis conditions: **Cage 4** concentration: 4 mM; TMC concentration: 6 mM, Reaction time: 10 min.

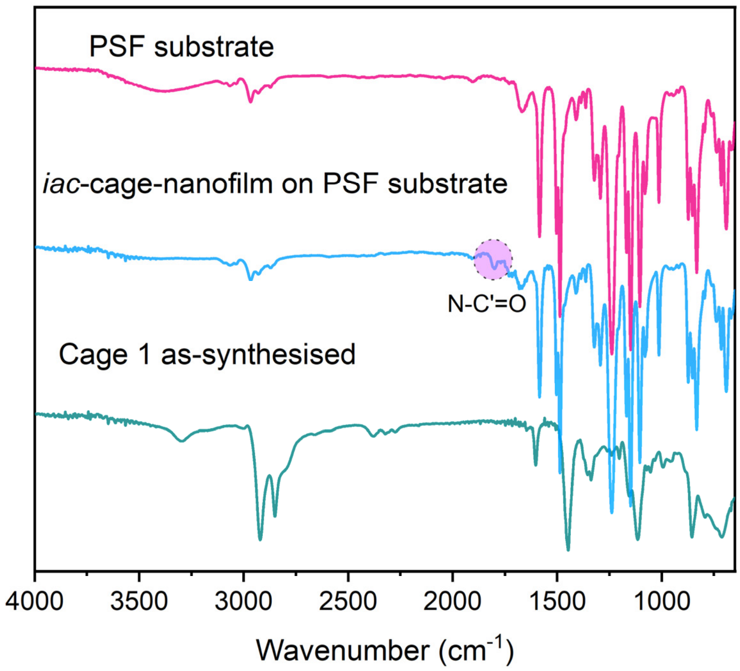

Supplementary Fig. 11 FT-IR spectra of the *iac*-cage-nanofilm and the corresponding porous substrate and Cage 1 as prepared. Synthesis conditions for the this nanofilm: **Cage 1** concentration: 4 mM; TMC concentration: 6 mM, Reaction time: 10 min. The appearance of the characteristic band of amide bond at around 1750 cm^{-1} in the FTIR spectrum of the *iac*-cage nanofilm verified the formation of amide linkages.

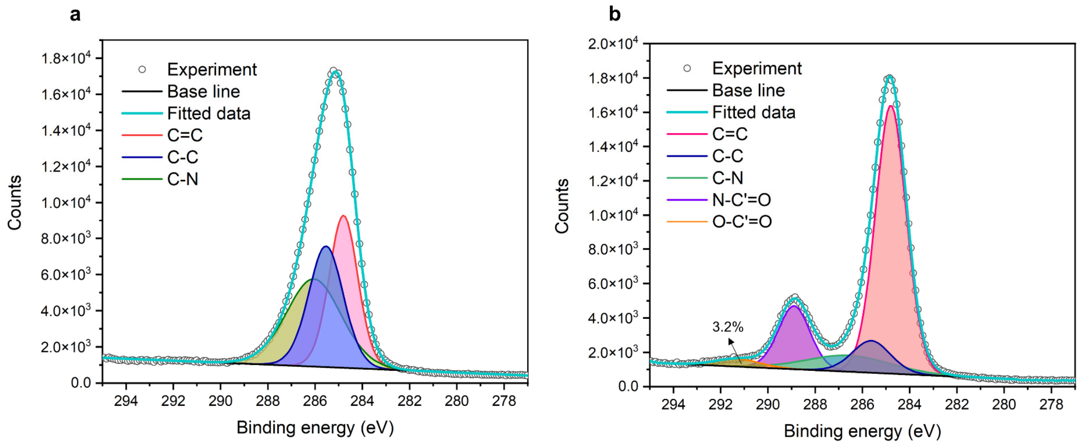

Supplementary Fig. 12 X-ray photoelectron spectroscopy C1*s* spectrum of a) powdered Cage 1 and b) the networked cage nanofilms. The cage nanofilm was prepared from 4 mM **Cage 1** and 6 mM TMC reacted for 10 min. The appearance of -N-C=O species in the C1*s* spectrum of cage nanofilm reveal the formation of amide linkages.

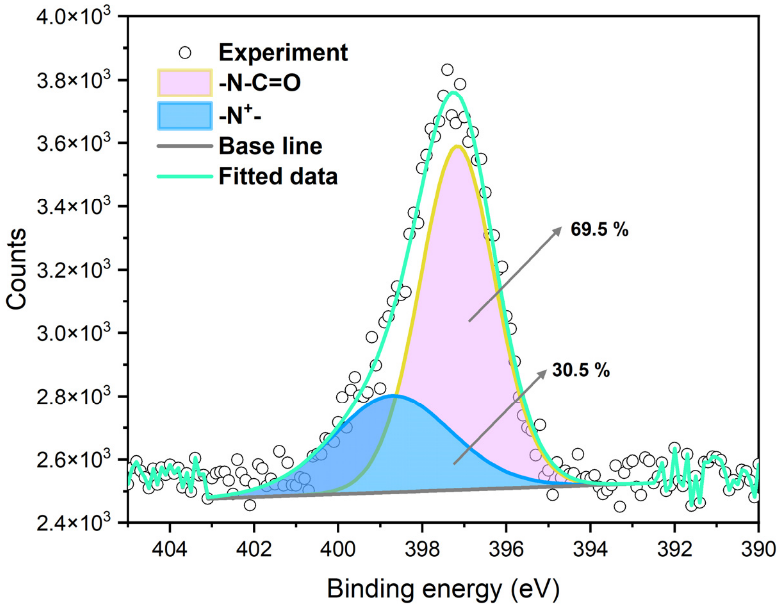

Supplementary Fig. 13 X-ray photoelectron spectroscopy N1*s* spectrum of the cage nanofilms. The cage nanofilm was prepared from 4 mM **Cage 1** and 6 mM TMC reacted for 10 min. The percentage of crosslinked -NH- (PC_{NH}) in the cage nanofilm can be calculated to be nearly 70 %.

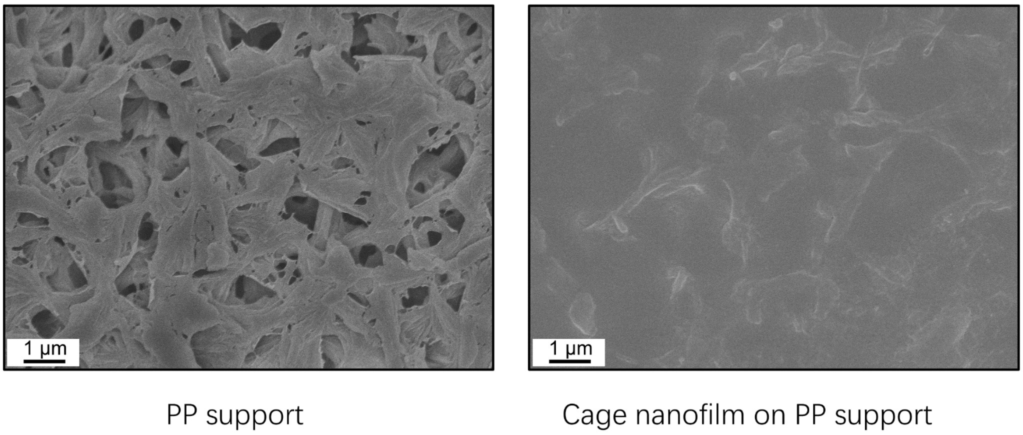

Supplementary Fig. 14 Networked cage nanofilm transferred onto polypropylene (PP) microfiltration membrane. The PP substrate is highly rough (left). All the pores of the PP support were obstructed by the smooth nanofilms, while the profiles of the substrates were still visible, revealing the dense, ultrathin and flexible features of the cage nanofilm. Synthesis conditions: **Cage 1** concentration: 4 mM; TMC concentration: 6 mM, Reaction time: 10 min.

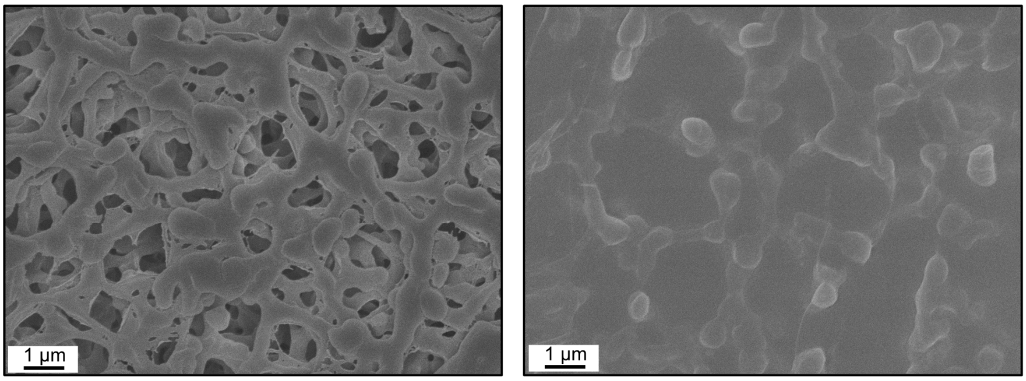

Cellulose support   Cage nanofilm on cellulose support

Supplementary Fig. 15 Networked cage nanofilm transferred onto cellulose microfiltration membrane (pore size: 0.22 μm). Similarly, the cellulose substrate is highly rough (left). All the pores of the cellulose support were obstructed by the smooth nanofilms, while the profiles of the substrates were still visible, revealing the dense, ultrathin and flexible features of the cage nanofilm. Synthesis conditions: **Cage 1** concentration: 4 mM; TMC concentration: 6 mM, Reaction time: 10 min.

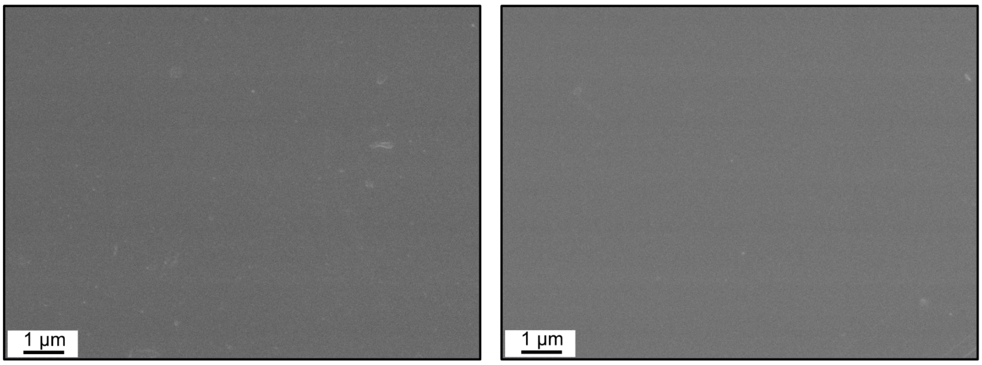

PSF support   Cage nanofilm on PSF support

Supplementary Fig. 16 Networked cage nanofilm transferred onto PSF ultrafiltration membrane (MWCO=10 K Da). The cage nanofilm was also transferred onto a dense ultrafiltration membrane. In contrast, the membrane surface became smoother and no defect was observed. Synthesis conditions: **Cage 1** concentration: 4 mM; TMC concentration: 6 mM, Reaction time: 10 min.

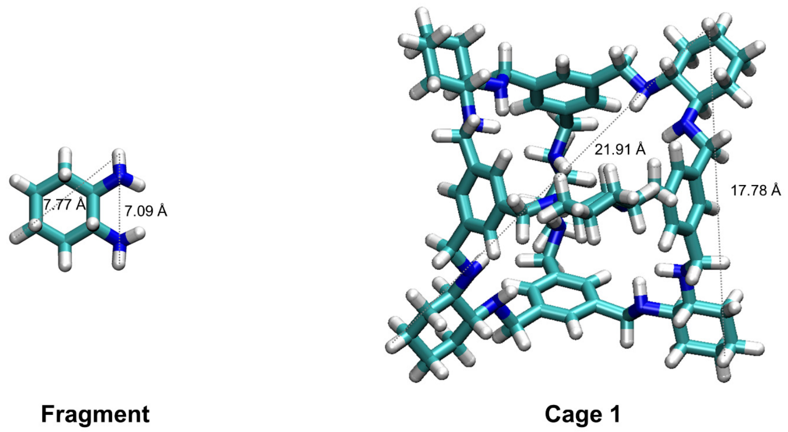

Supplementary Fig. 17 Molecular size calculation of Cage 1 and the fragment. Accordingly, the cage molecule has a maximum size of 21.91 Å that is approximately 3 times that of the Fragment. In other words, the cage molecule possesses a cluster-scale dimension.

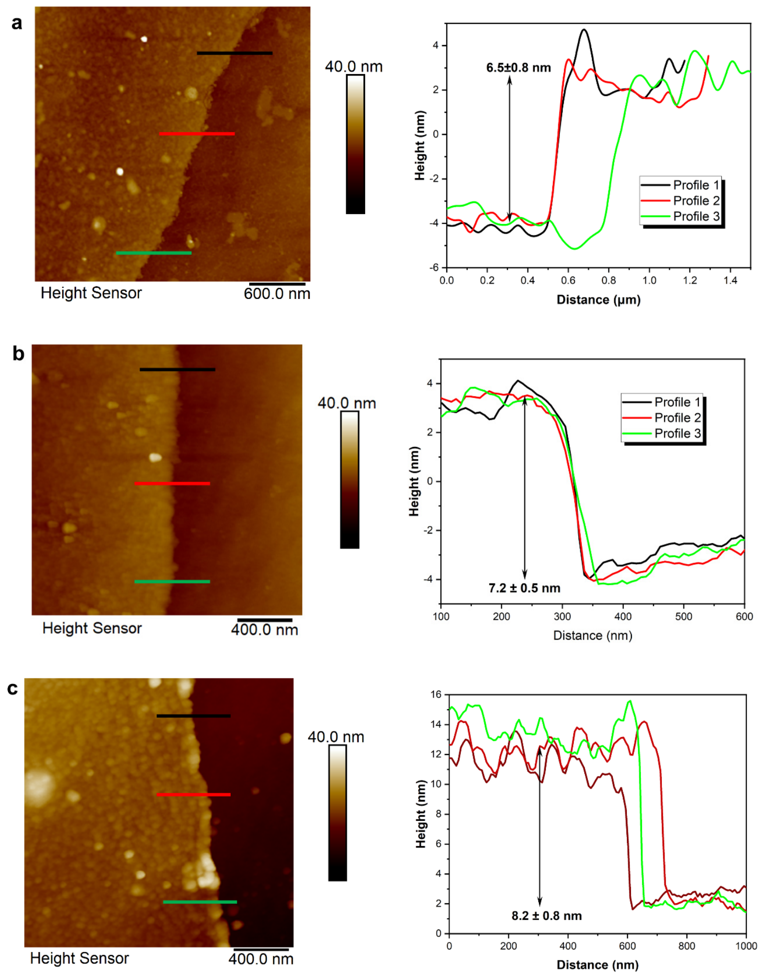

Supplementary Fig. 18 AFM topographic images and height profiles of the cage nanofilm prepared from Cage 1 with reaction time of a) 2 min, b) 5 min and c) 10 min. Thickness slightly increased with reaction time.

Supplementary Table 1. Surface roughness of cage nanofilms.

Cage nanofilm (Cage-concentration-reaction time)	Average roughness (nm)
Cage1-4 mM-2 min	0.45±0.03
Cage1-4 mM-5 min	0.60±0.04
Cage1-4 mM-10 min	0.80±0.05
Cage2-4 mM-10 min	0.95±0.04
Cage3-4mM-10 min	0.55±0.05
Cage4-4mM-10 min	0.80±0.04

At least three 2×2 μm^2 AFM scan sizes were used to calculate the average roughness.

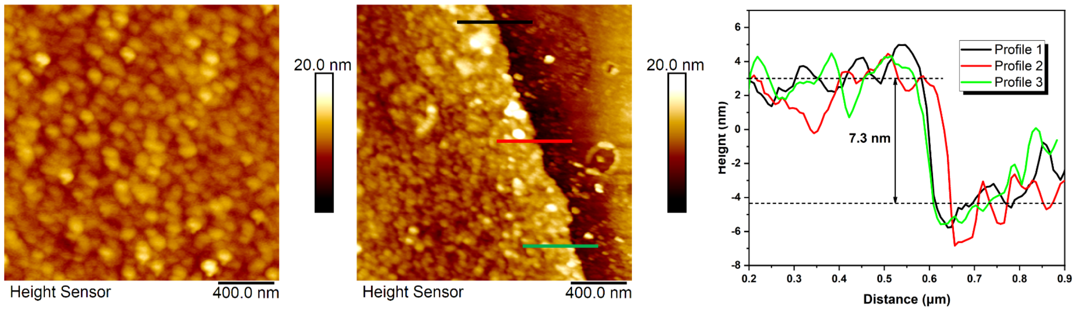

Supplementary Fig. 19 AFM topographic images and height profiles of the cage nanofilm prepared with Cage 2. Synthesis conditions: **Cage 2** concentration: 4 mM; TMC concentration: 6 mM, Reaction time: 10 min.

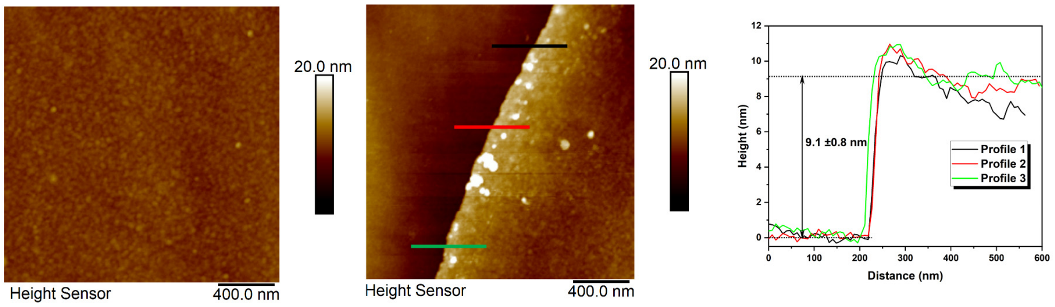

Supplementary Fig. 20 AFM topographic images and height profiles of the cage nanofilm prepared with Cage 3. Synthesis conditions: **Cage 3** concentration: 4 mM; TMC concentration: 6 mM, Reaction time: 10 min.

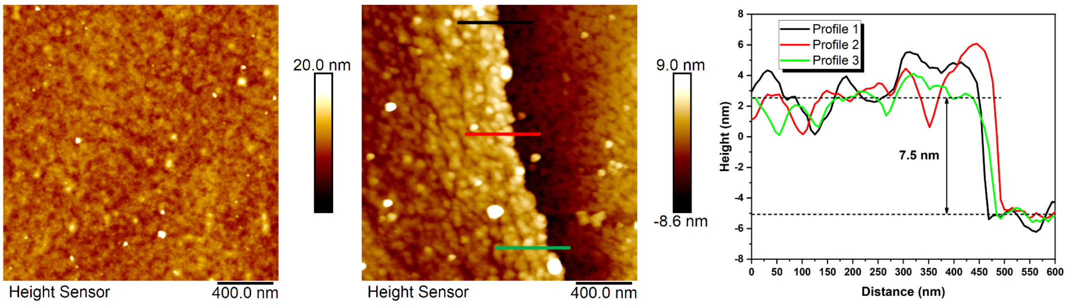

Supplementary Fig. 21 AFM topographic images and height profiles of the cage nanofilm prepared with Cage 4. Synthesis conditions: **Cage 4** concentration: 4 mM; TMC concentration: 6 mM, Reaction time: 10 min.

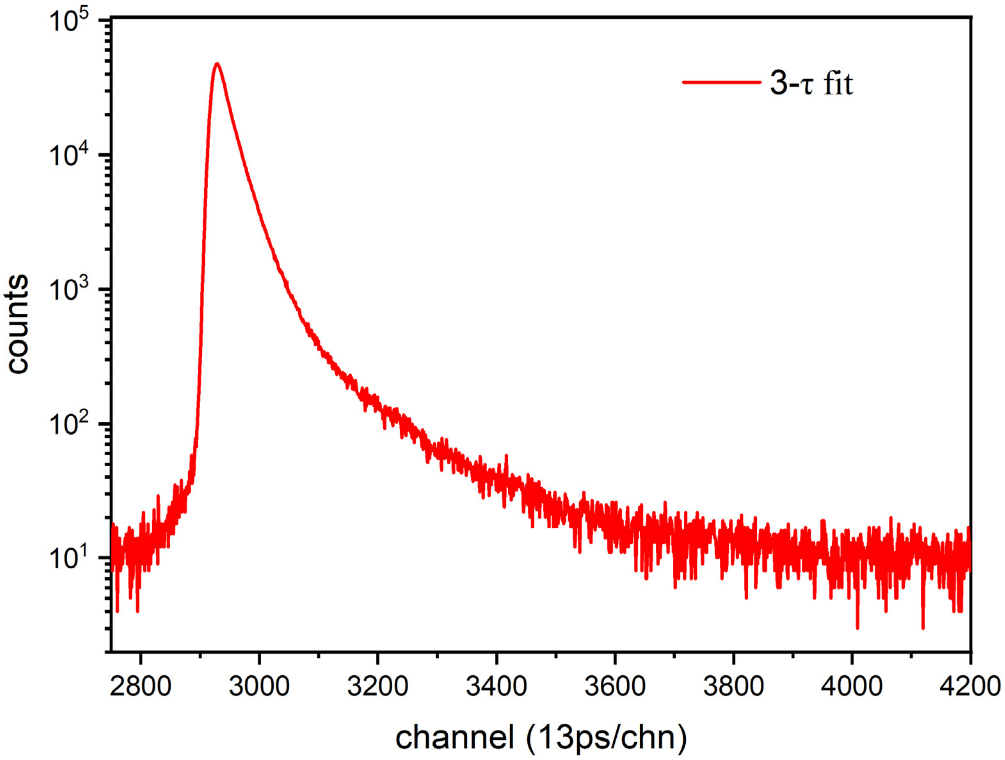

Supplementary Fig. 22 Measured positron lifetime spectrum and the 3-component fitting curve for power of crosslinked Cage 1.

Supplementary Table 2. Summary of PALS parameters.

τ1 (ns)	error (ns)	I1 (%)	error (%)	τ2 (ns)	error (ns)	I2 (%)	error (%)	τ3 (ns)	error (ns)	I3 (%)	error (ns)
0.2099	0.0047	44.0	1.6	0.4247	0.0082	48.6	1.6	1.803	0.016	7.39	0.26

The τ1, τ2, and τ3 represent lifetime components, I1, I2, and I3 represent their intensities.

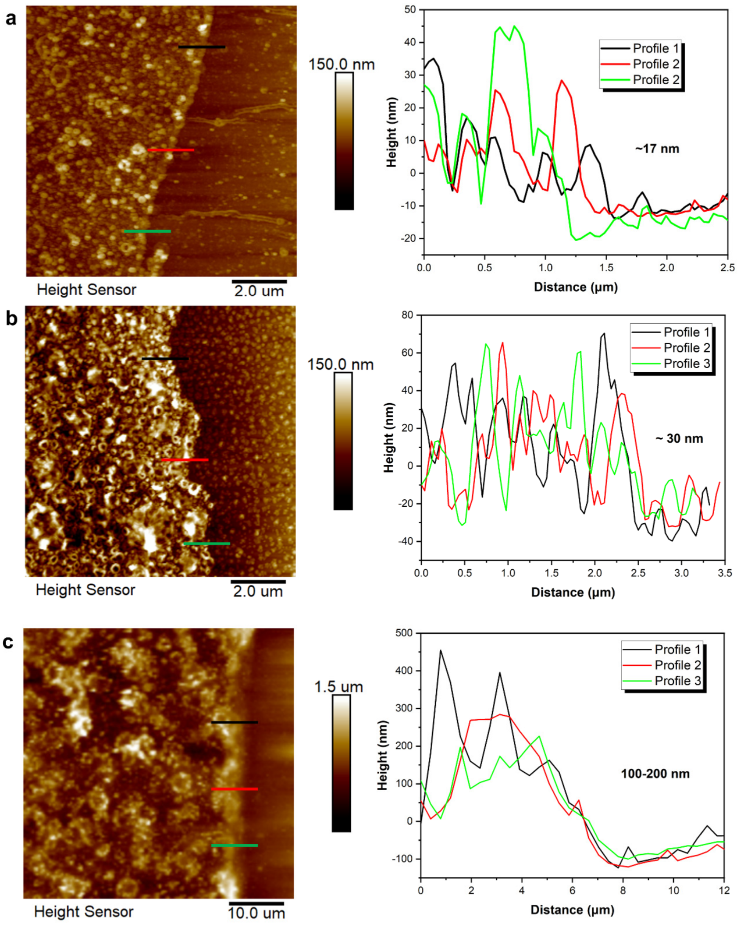

Supplementary Fig. 23 AFM topographic images and height profiles of the nanofilm prepared from (R, R)-1,2-diaminocyclohexane (fragment) and TMC with a reaction time of a) 2 min, b) 5 min and c) 10 min. Surface morphology and thickness varied obviously with reaction time.

Supplementary Table 3. Surface roughness of the control nanofilms.

Control nanofilm (Diamine-concentration-reaction time)	Average roughness (nm)
Diamine-4 mM-2 min	10.0±2.5
Diamine-4 mM-5 min	21.0±10.3
Diamine-4 mM-10 min	52.0±23.5

At least three 2×2 μm^2 AFM scan sizes were used to calculate the average roughness.

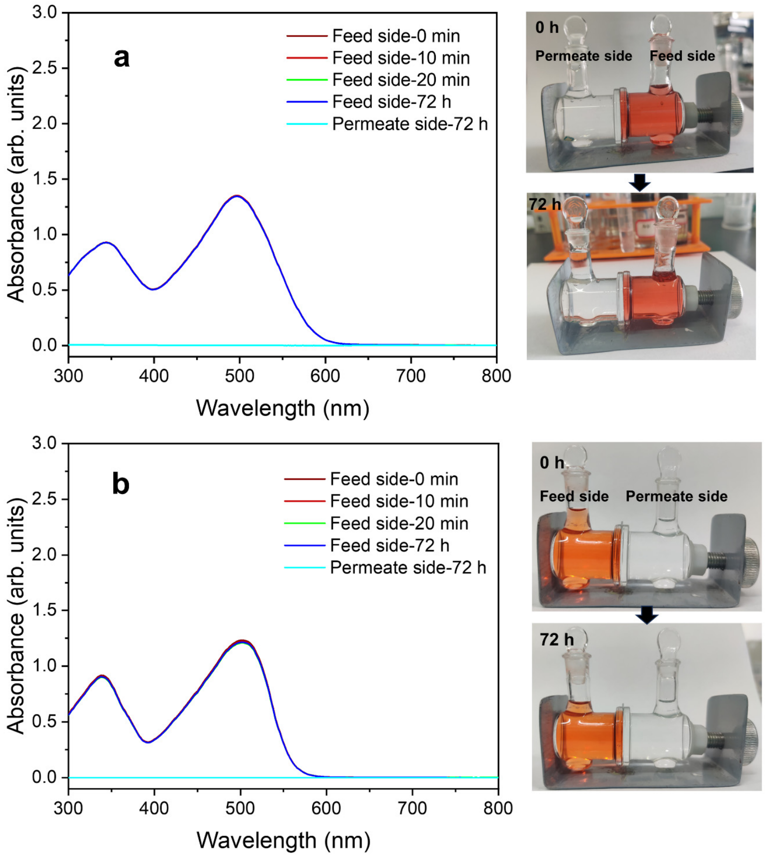

Supplementary Fig. 24 Dye adsorption test using a Valia-Chien diffusion cell. Solvent: a) water; b) methanol. Membrane samples of cage composite membrane with a diameter of 2 cm were fixed between the diffusion cell. One chamber was filled with a 100 ppm Congo red dye solution, using either water or methanol as the solvent, while the other chamber was filled with pure solvent. The dye concentration in the solution was monitored using UV-vis absorption. No noticeable variation in UV-vis absorption was observed in the feed chamber, and the permeate chamber remained colorless even after 72 hours. These findings confirm that the membrane did not absorb the dyes.

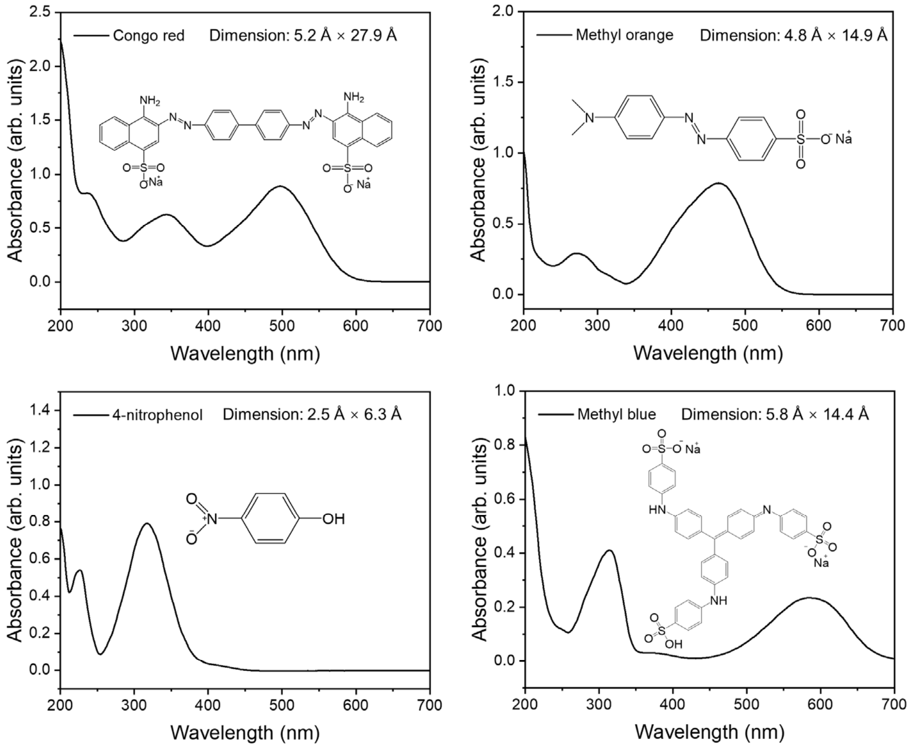

Supplementary Fig. 25 Molecular structure and UV/vis absorbance of dyes with varying dimensions.

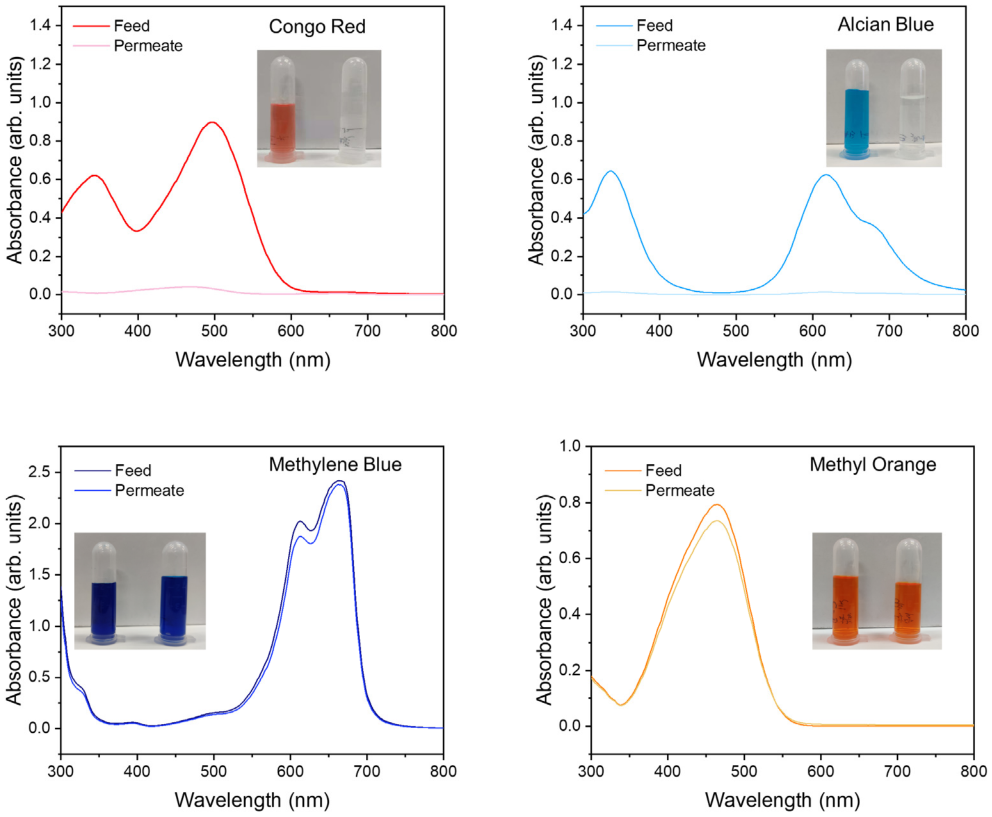

Supplementary Fig. 26 UV-vis absorption spectra of dyes in feed and permeate. The rejection order is Congo Red (99.2) > Alcian Blue (98.3) > Methyl Orange (7.3) > Methylene Blue (1.4).

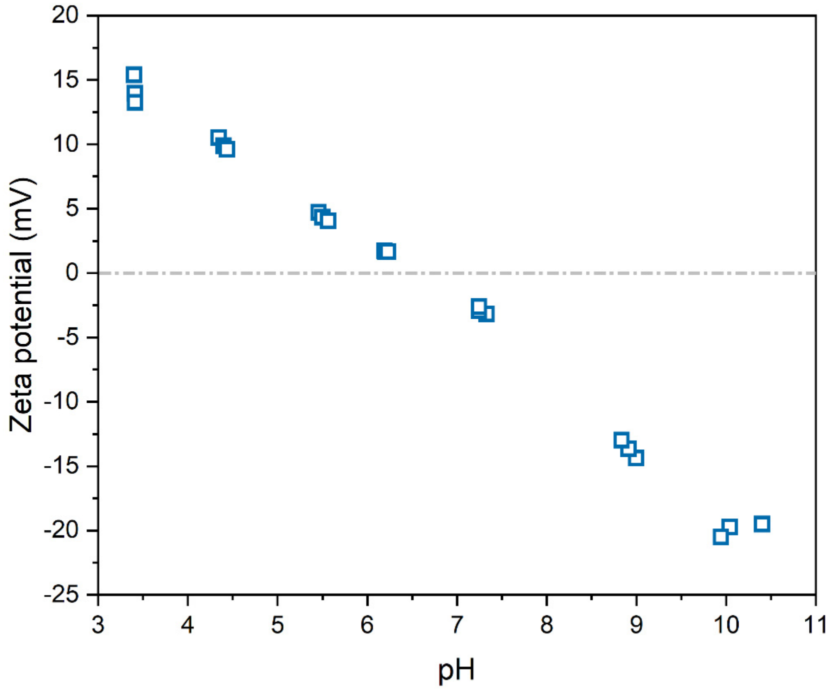

Supplementary Fig. 27 Surface zeta potential of the cage composite membrane. The membrane surface is slightly negatively charged at around pH=7.

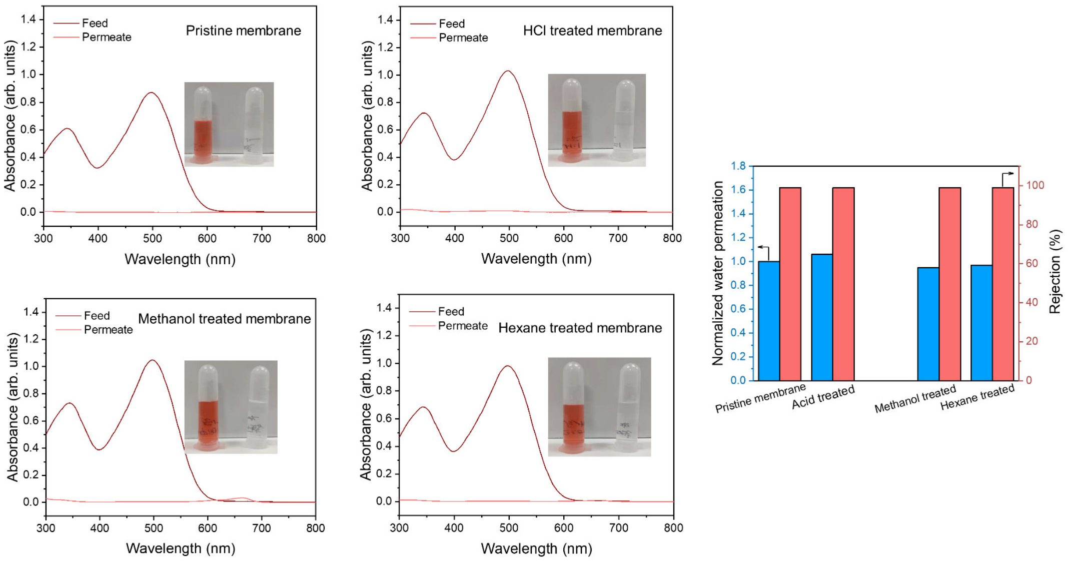

Supplementary Fig. 28 Stability performance of cage composite membrane immersing in strong acid aqueous solutions, and polar/nonpolar organic solvents.

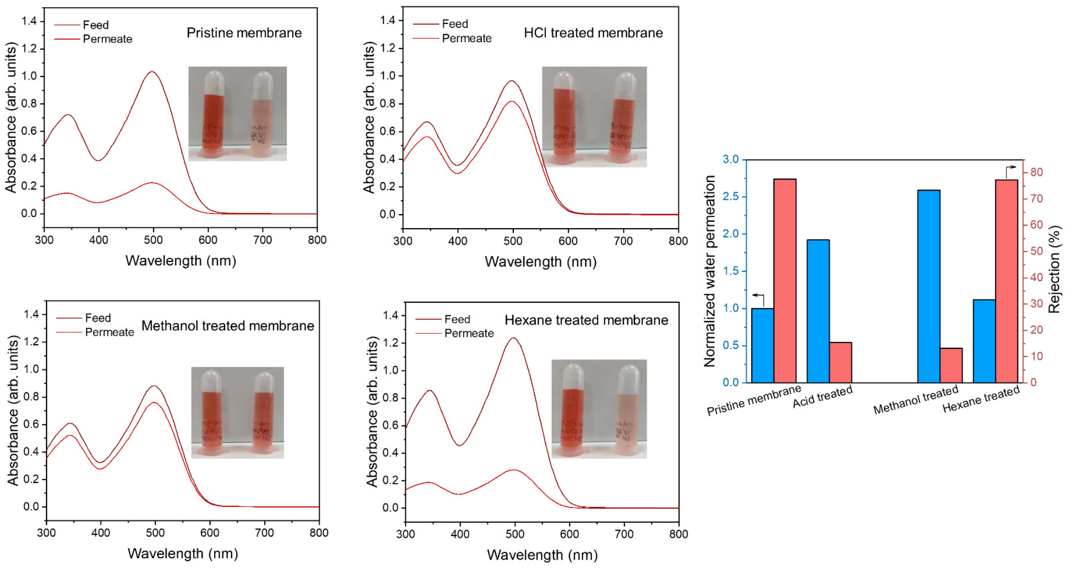

Supplementary Fig. 29 Stability performance of the spin-coated Cage 1 composite membrane immersing in strong acid aqueous solutions, and polar/nonpolar organic solvents.

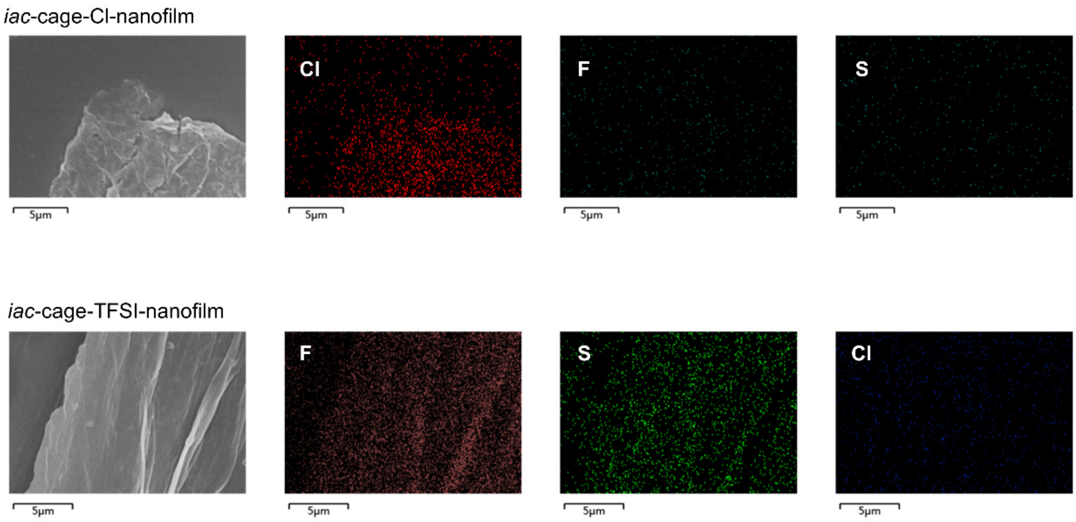

Supplementary Fig. 30 EDS element analysis for nanofilm bearing counterions of Cl^- and $TFSI^-$.

Supplementary Table 4. Surface atomic composition of nanofilms with various counterions

Nanofilm	Atomic composition from EDS					
	C	N	O	Cl	F	S
iac-cage-Cl	70.7	7.9	18.8	2.6	ND	ND
iac-cage-TFSI	64.6	6.5	15.6	0.01	10.1	3.2
iac-cage-azo	72.2	8.5	19.3	0.02	ND	ND

ND: not detected.

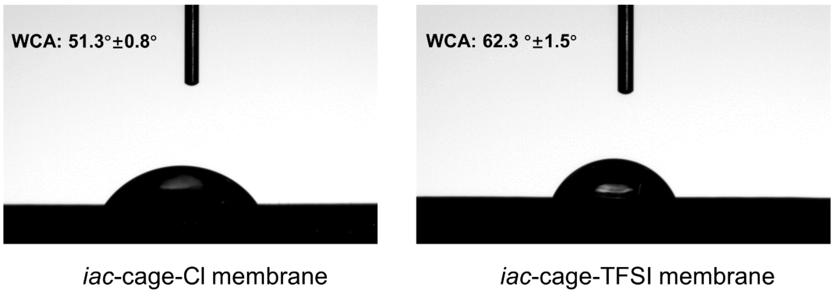

Supplementary Fig. 31 Water contact angle measurements. The *iac*-cage-TFSI membrane is more hydrophobic than the *iac*-cage-Cl-membrane due to the more hydrophobic nature of $TFSI^-$ counterion.

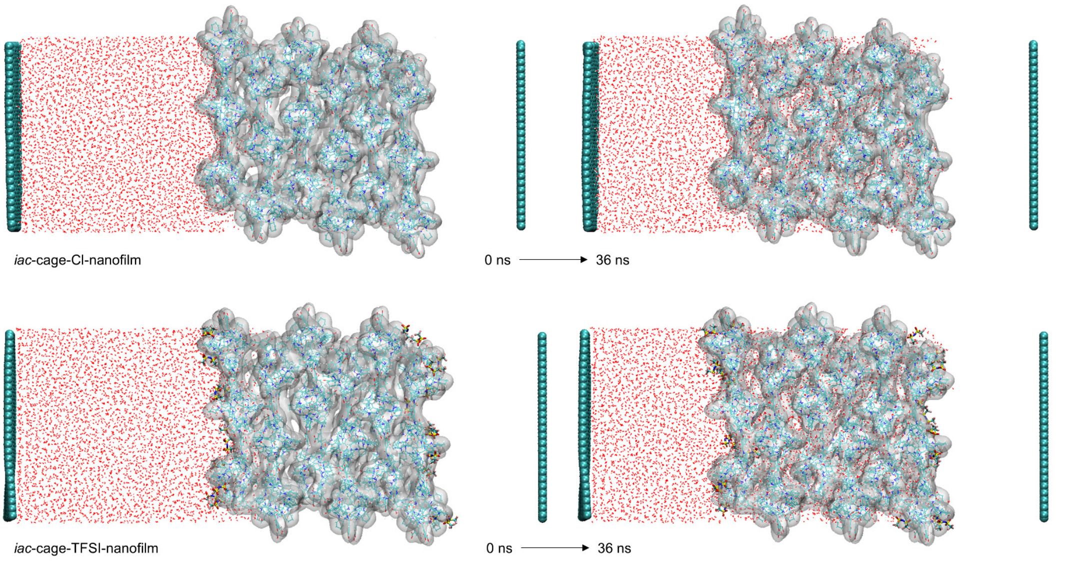

Supplementary Fig. 32 Snapshots (0 ns and 36 ns) of water molecules diffusion in the nanofilms of *iac*-cage-Cl and *iac*-cage-TFSI.

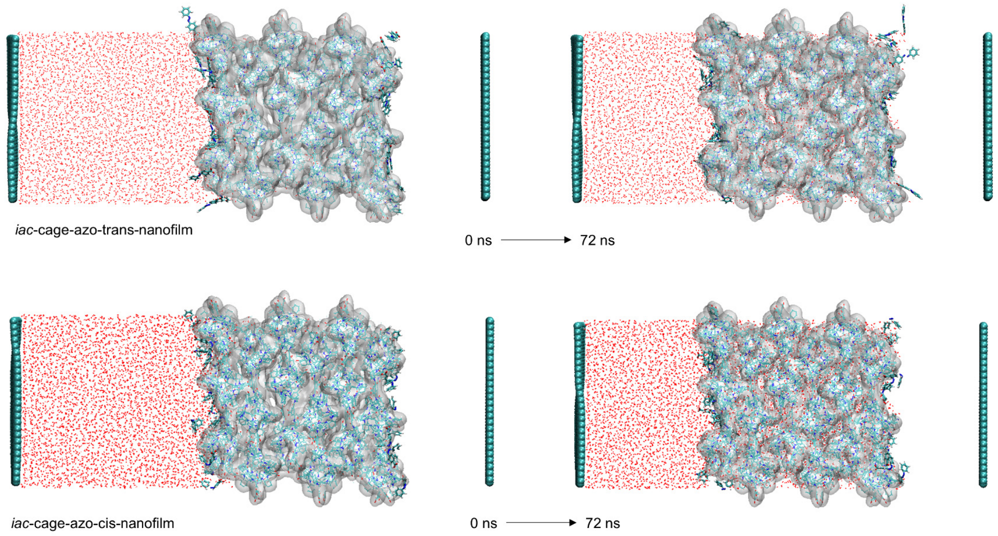

Supplementary Fig. 33 Snapshots (0 ns and 72 ns) of water molecules diffusion in the nanofilms of *iac*-cage-azo-trans and *iac*-cage-azo-cis.

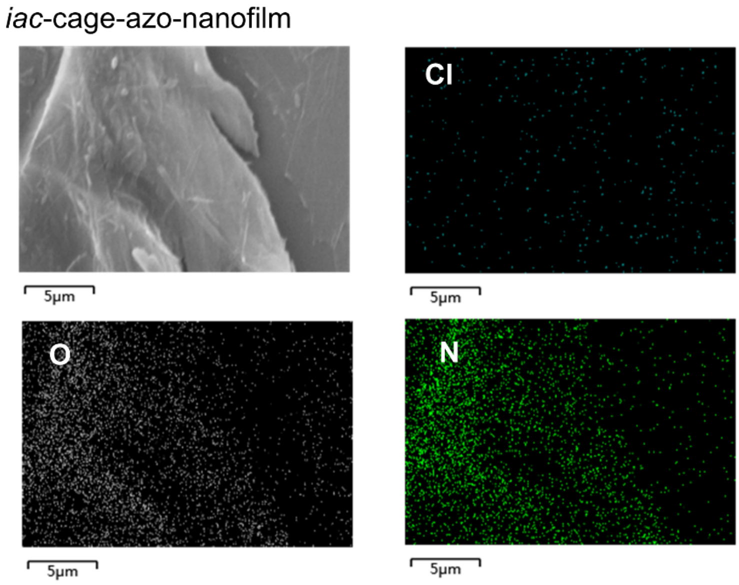

Supplementary Fig. 34 EDS element analysis for nanofilm bearing counterions of azo⁻.

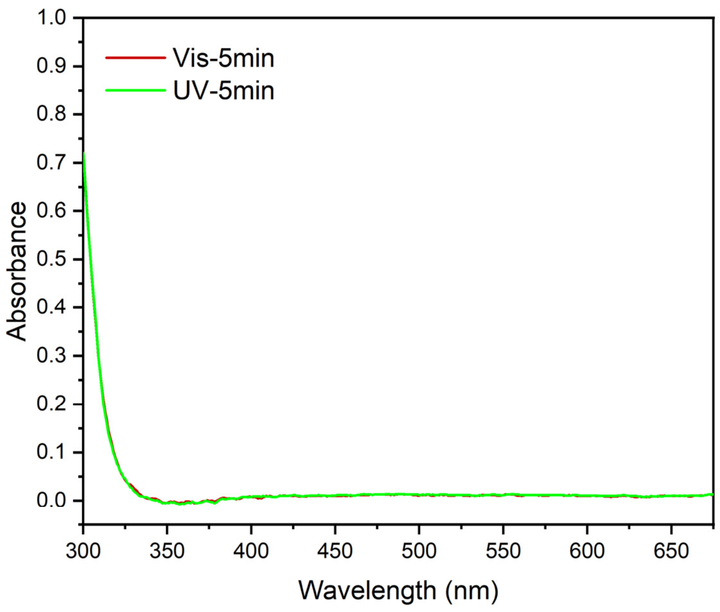

Supplementary Fig. 35 UV/Vis spectra of the *iac*-cage-Cl membrane at 298 K, using UV and Vis irradiation (5min). No obvious UV-Vis absorbance variation can be detected.

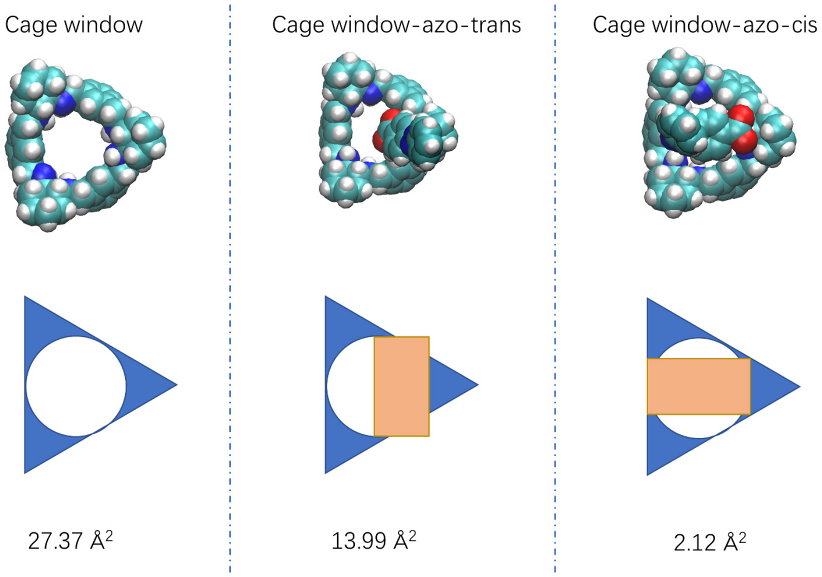

Supplementary Fig. 36 Pore aperture demonstration and projected area calculation. Here, **Cage 1** equipped with cis-azo displays a much smaller projected area than that with trans-azo.

Supplementary References

[1] Liu, C. et al. Interfacial polymerization at the alkane/ionic liquid interface. *Angew. Chem. Int. Ed.* **133**, 2–10 (2021).

[2] Frisch, A., gaussian 09W Reference. Wallingford, USA, 25p, 470 (2009).

[3] Grimme, S., Antony, J., Ehrlich, S., Krieg, H. A. consistent and accurate ab initio parametrization of density functional dispersion correction (DFT-D) for the 94 elements H-Pu. *J. Chem. Phys.* **132**, 154104 (2010).

[4] Walker, M., Harvey, A. J., Sen, A., Dessent, C. E. Performance of M06, M06-2X, and M06-HF density functionals for conformationally flexible anionic clusters: M06 functionals perform better than B3LYP for a model system with dispersion and ionic hydrogen-bonding interactions. *J. Phys. Chem. A* **117**, 12590–12600 (2013).

[5] Marenich, A. V., Cramer, C. J., Truhlar, D. G. Performance of SM6, SM8, and SMD on the SAMPL1 test set for the prediction of small-molecule solvation free energies. *J. Phys. Chem. B* **113**, 4538–4543 (2009).

[6] Haranczyk, M., Rycroft, C., Martin, R., Willems, T. Zeo++: High-throughput analysis of crystalline porous materials, v0. 2.2. Lawrence Berkeley National Laboratoy, Berkeley, 2012á SearcháPubMed 2012.

[7] Van Der Spoel, D. et al. GROMACS: fast, flexible, and free. *J. Comput. Chem.* **26**, 1701–1718 (2005).

[8] Sprenger, K., Jaeger, V. W., Pfaendtner, J. The general AMBER force field (GAFF) can accurately predict thermodynamic and transport properties of many ionic liquids. *J. Phys. Chem. B* **119**, 5882–5895 (2015).

[9] Lu. T. Sobtop, Version [1.0(dev3.1)], http://sobereva.com/soft/Sobtop (accessed on 20-Mar-2023).

[10] Duchstein, P., Neiss, C., Görling, A., Zahn, D. Molecular mechanics modeling of azobenzene-based photoswitches. *J. Mol. Model.* **18**, 2479–2482 (2012).

[11] Lu, T., Chen, F. Multiwfn: A multifunctional wavefunction analyzer. *J. Comput. Chem.* **33**, 580–592 (2012).

[12] Humphrey, W., Dalke, A., Schulten, K. VMD: visual molecular dynamics. *J. Mol. Graph.* **14**, 33–38 (1996).

[13] Liu, Q. et al. Molecular dynamics simulation of water-ethanol separation through monolayer graphene oxide membranes: Significant role of O/C ratio and pore size. *Sep. Purif. Technol.* **224**, 219–226 (2019).
