# Supplementary material for: Sub-8 nm networked cage nanofilm with tunable nanofluidic channels for adaptive sieving
Source: Nat Commun. 2024 Mar 20;15:2478. doi: 10.1038/s41467-024-46809-4 (PMC10954766; doi:10.1038/s41467-024-46809-4)
Supplement: Supplementary file 3 — Description of Additional Supplementary Files [file 41467_2024_46809_MOESM3_ESM.pdf]

<u>Description of Additional Supplementary Files</u>

Supplementary Movie Legends:

Supplementary Movie 1: Self-assembly of the amphiphilic Cage 1 at the O/W interface.

Supplementary Movie 2: Water molecules diffusion in the iac-cage-Cl-nanofilm.

Supplementary Movie 3: Water molecules diffusion in the iac-cage-TFSI-nanofilm.

Supplementary Movie 4: Water molecules diffusion in the iac-cage-azo-transnanofilm.

Supplementary Movie 5: Water molecules diffusion in the iac-cage-azo-cis-nanofilm.
